# Neural substrates underlying progressive micrographia in Parkinson's disease

**DOI:** 10.1002/brb3.1669

**Published:** 2020-06-18

**Authors:** Shigenori Kanno, Mayumi Shinohara, Kasumi Kanno, Yukihiro Gomi, Makoto Uchiyama, Yoshiyuki Nishio, Toru Baba, Yoshiyuki Hosokai, Atsushi Takeda, Hiroshi Fukuda, Etsuro Mori, Kyoko Suzuki

**Affiliations:** ^1^ Department of Behavioural Neurology and Cognitive Neuroscience Tohoku University Graduate School of Medicine Sendai Japan; ^2^ Department of Occupational Therapy International University of Health and Welfare Narita Japan; ^3^ Department of Speech, Language, and Hearing Sciences Niigata University of Health and Welfare Niigata Japan; ^4^ Department of General Psychiatry Tokyo Metropolitan Matsuzawa Hospital Setagaya Japan; ^5^ Department of Neurology Sendai Nishitaga Hospital Sendai Japan; ^6^ Department of Radiological Science International University of Health and Welfare Otawara Japan; ^7^ Department of Nuclear Medicine and Radiology Institute of Development, Aging and Cancer Tohoku University Sendai Japan; ^8^ Division of Radiology Tohoku Medical and Pharmaceutical University Sendai Japan; ^9^ Department of Behavioural Neurology and Cognitive Neuropsychiatry Osaka University United Graduate School of Child Development Suita Japan

**Keywords:** anterior cingulate cortex, micrographia, Parkinson's disease, positron emission tomography, supplementary motor area, visual cortex

## Abstract

**Introduction:**

The neural substrates associated with the development of micrographia remain unknown. We aimed to elucidate the neural substrates underlying micrographia in Parkinson's disease (PD) patients.

**Methods:**

Forty PD patients and 20 healthy controls underwent handwriting tests that involved free writing and copying. We measured the size of each letter and the resting cerebral glucose metabolic rate of the PD patients and another group of age‐ and sex‐matched 14 healthy controls (HCs), who had not participated in the writing tests, using resting‐state 18F‐fluorodeoxyglucose positron emission tomography.

**Results:**

In the PD patients, the prevalence of consistent micrographia (CM) associated with free writing was 2.5% for both tasks. Alternatively, the prevalence of progressive micrographia (PM) was 15% for free writing and 17.5% for copying. In the PD patients, there was no significant difference in the letter sizes between these tasks, whereas the variability of the letter sizes for copying was significantly different from that for free writing. The means and decrements in letter sizes in either task were not significantly correlated with the severity of brady/hypokinesia in the PD patients. For free writing, the PD patients with PM showed glucose hypometabolism in the anterior part of the right middle cingulate cortex, including the rostral cingulate motor area, compared with those without PM. For copying, the PD patients with PM showed glucose hypometabolism in the right superior occipital gyrus, including V3A, compared with those without PM.

**Conclusions:**

These findings suggest that PM in free writing in PD patients is caused by the difficulty of monitoring whether the actual handwriting movements are desirable for maintaining letter size during self‐paced handwriting. By contrast, PM in copying in PD patients is evoked by a lack of visual information about the personal handwriting and hand motions that are used as cues for maintaining letter sizes.

## Graphical abstract

In Parkinson's disease, progressive micrographia in free writing is caused by dysfunction of the cingulate motor area and presupplementary motor area. By contrast, progressive micrographia in copying is evoked by dysfunction of the visuospatial area.

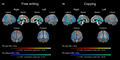

## 1 INTRODUCTION

Micrographia is characterized by either overall small handwriting (consistent micrographia: CM) or a serial reduction in handwriting size (progressive micrographia: PM; Kim, Lee, Park, Lee, & Na, 2005; Pick, 1903; Wilson, 1925). Micrographia is characteristic of patients with Parkinson's disease (PD) (McLennan, Nakano, Tyler, & Schwab, 1972) but has also been observed in patients with other neurodegenerative diseases, such as progressive supranuclear palsy or corticobasal syndrome, brain tumors, cerebrovascular disease, head trauma, hypoxic encephalopathy, and central nervous system inflammatory diseases, such as systemic lupus erythematosus, multiple sclerosis, or anti‐N‐methyl‐D‐aspartate receptor encephalitis (Ishihara et al., 2006; Lewitt, 1983; Ling, Massey, Lees, Brown, & Day, 2012; Martínez‐Vila, Artieda, & Obeso, 1988; Münchau et al., 2000; Kadoya, Kadoya, Onoue, Ikewaki, & Kaida, 2015; Sakurai et al., 2013; Scolding & Lees, 1994; Wagle Shukla et al., 2012; Yoshida, Yamadori, & Mori, 1989). Based on symptoms observed in patients with PD, micrographia may constitute an aspect of brady/hypokinesia (Wagle Shukla et al., 2012). However, this situation does not necessarily apply to all patients with PD because micrographia sometimes manifests as the initial symptom (McLennan et al., 1972). Many studies have also reported the presence of micrographia without other parkinsonian features, including brady/hypokinesia, due to focal lesions (Inzelberg, Plotnik, Harpaz, & Flash, 2016; Ishihara et al., 2006; Lewitt, 1983; Martínez‐Vila et al., 1988; Scolding & Lees, 1994; Yoshida et al., 1989). Therefore, the association between micrographia and brady/hypokinesia remains unclear.

The letter sizes of patients with PD can be easily altered by visual conditions (Kim et al., 2005; Ondo & Satija, 2007). Ondo and Satija (2007) noted that the severity of micrographia in patients with PD in the off‐medication state was reduced by closing the eyes (visual withdrawal) and mentioned that there is symptomatic commonality between micrographia and gate freezing. Kim et al. (2005) reported that CM and PM in patients with PD are more commonly observed in copying than in free writing tasks. Regardless of the underlying pathophysiological mechanism involved in PD with micrographia, the presence of visual cues or feedback can interrupt the process needed to continue writing letters of the same size.

Recent studies using functional MRI have been conducted to elucidate the neural activity and connectivity modulations related to micrographia in PD patients. Wu et al. (2016) suggested that CM is related to dysfunction of the basal ganglia motor circuit (the left posterior putamen, left thalamus, and left caudal supplementary motor area [SMA]), which is linked to bradykinesia in PD patients. These authors also noted that PM is associated with a disconnection among the presupplementary motor area (pre‐SMA), rostral cingulate motor area (CMAr), and cerebellum in addition to dysfunction of the basal ganglia motor circuit. Nackaerts et al. found that patients with PD exhibited weaker connectivity between the MT/V5 area and superior parietal lobule (SPL) than did healthy controls during handwriting tasks (Nackaerts, Michely, et al., 2018). These authors also demonstrated that the connectivity between visual and motor areas to the SPL is strengthened by visual cueing in patients with PD as well as in healthy controls, even though visual cueing does not improve handwriting performance (Nackaerts, Michely, et al., 2018). It has been reported that dysfunction of the right dorsolateral occipital and right posterior parietal areas is strongly associated with impairment in visuospatial attention in patients with PD without dementia (Abe et al., 2003). Their findings indicate that the basal ganglia motor circuit and connectivity among the motor areas and visuo‐parietal coupling are associated with maintaining letter sizes in handwriting tasks in PD patients with micrographia. Therefore, we hypothesized that the appearance of micrographia might be altered depending on the regional differences in dysfunction of the basal ganglia motor circuit and visuospatial areas.

In the present study, we attempted to investigate the occurrence mechanism of micrographia in Parkinson's disease patients using ^18^F‐fluorodeoxyglucose positron emission tomography (FDG‐PET) because FDG‐PET may be more sensitive than functional MRI in detecting the affected brain lesions in patients with PD, which are similar to those in patients with several other neurological or psychiatric disorders, such as consciousness disturbance, Alzheimer's disease, seizure, and mood disorder (Fu et al., 2018; Kamm et al., 2018; Staffaroni et al., 2017; Stender et al., 2014). Additionally, to the best of our knowledge, no studies have been conducted to determine which brain regions exhibit resting‐state hypo‐ or hypermetabolism in patients with PD with micrographia.

## 2 MATERIALS AND METHODS

### 2.1 Participants

The participants included forty (26 women/14 men) right‐handed patients with PD and 20 right‐handed healthy controls (HCs) who were matched by age, sex, educational attainment, and Mini‐Mental State Examination (MMSE) score. All patients were recruited from Tohoku University Hospital and were diagnosed by board‐certified neurologists based on the UK Parkinson's Disease Society Brain Bank Criteria (Gibb & Lees, 1988). The HCs were recruited from local communities via an advertisement. Patients' motor symptoms were evaluated using Hoehn‐Yahr staging (Hoehn & Yahr, 1967) and the motor part of the Unified Parkinson's Disease Rating Scale (UPDRS) (Fahn & Elton, 1987). The UPDRS motor scores were recorded while patients were in the on‐medication state. The inclusion criteria for patients in this study were as follows: (1) >55 years of age, with the age at onset being above 40 years; (2) a Hoehn‐Yahr stage between 1 and 3; and (3) a score of 24 or higher on the Mini‐Mental State Examination (MMSE). The exclusion criteria were as follows: (1) a medical history of a disease of the central nervous system not directly related to Parkinson's disease, such as stroke, head injury, or epilepsy; (2) a concurrent psychiatric illness, such as schizophrenia or manic depression; and (3) a documented or suspected history of drug abuse and/or alcoholism, diabetes mellitus, and major abnormalities on brain MRI scans, such as cerebral infarction or tumor. The demographic characteristics of the participants are shown in Table 1.

**TABLE 1:** Demographic characteristics of the subjects

Variables	PD	HCs	*p*‐Value
Number	40	20	
Age (years)	68.0 ± 6.6	69.4 ± 4.8	.403
Sex (women/men)	26/14	17/3	.105
Education attainment (years)	12.0 ± 1.9	12.7 ± 2.3	.258
MMSE score	27.8 ± 2.0	28.8 ± 1.9	.059
Hoehn‐Yahr stage	2.81 ± 0.51		
UPDRS motor score	19.0 ± 8.0		
Brady/hypokinesia score	6.5 ± 3.5		
Disease duration (years)	8.1 ± 4.6		
Levodopa equivalent dose (mg/day)	516 ± 280		

Note

Data are given as mean ± *SD* except for sex. Brady/hypokinesia score = the sum of scores finger taps, hand movements, rapid alternating movements of hands, leg agility, and body brady/hypokinesia from UPDRS motor part. Levodopa equivalent dose = regular levodopa dose 1 + slow‐release levodopa × 0.75 + bromocriptine × 10 + apomorphine × 10 + ropinirole × 20 + pergolide × 100 + pramipexole × 100 + [regular levodopa dose + (slow‐release levodopa × 0.75)] × 0.2 if taking entacapone.

The two‐sample *t* test was used except for sex (the chi‐square test).

Abbreviations: HCs, healthy controls; MMSE, Mini‐Mental State Examination; PD, Parkinson's disease; UPDRS, Unified Parkinson's Disease Rating Scale.

This study was conducted in accordance with the Declaration of Helsinki, and the protocol was approved by Tohoku University. Prior to the beginning of this study, written consent was obtained from each participant.

### 2.2 Evaluation of micrographia

**FIGURE 1:**
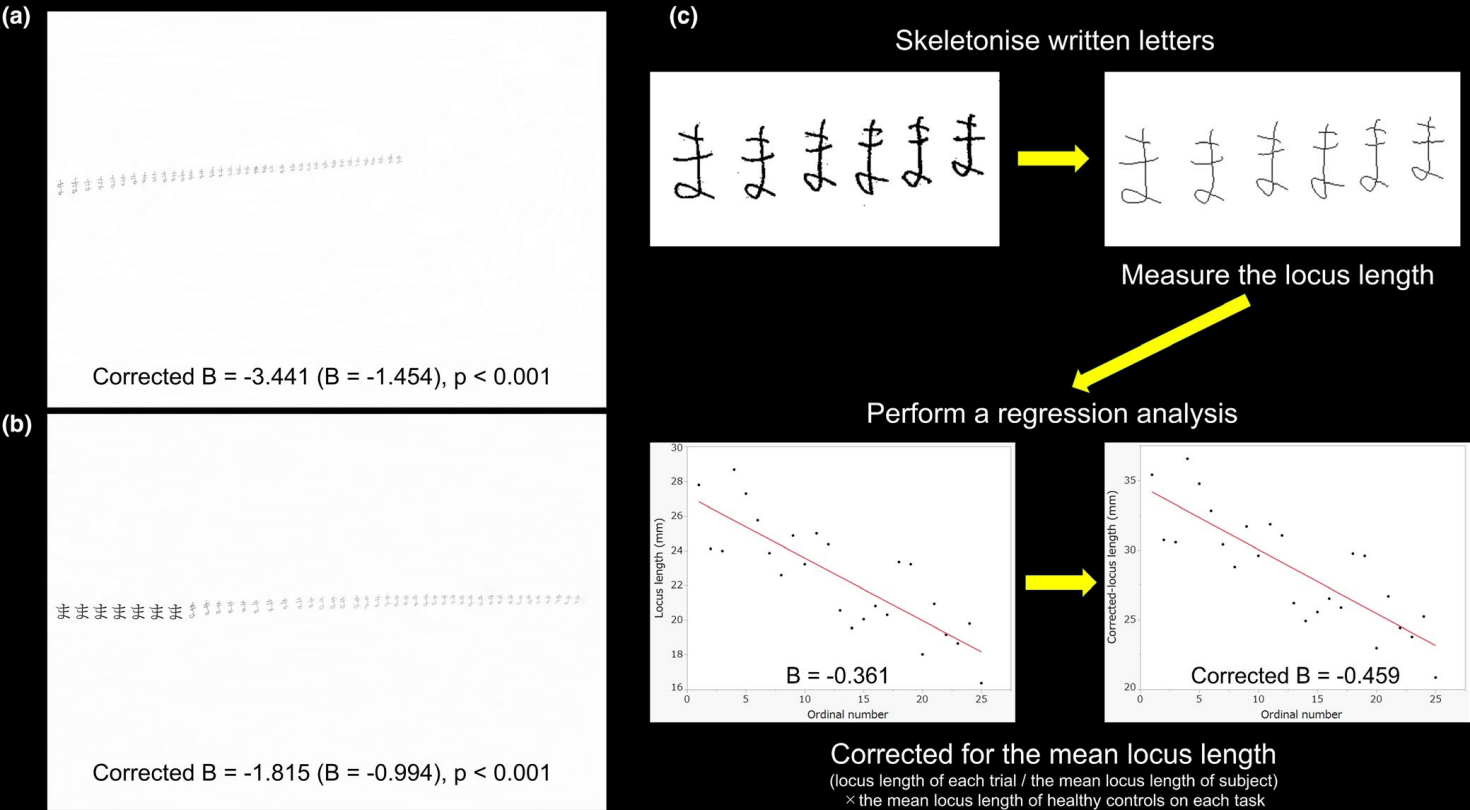
(a) A sample case showing progressive micrographia (PM) in the free writing task in patients with Parkinson's disease (PD). In this case, the *B* value (the slope of the regression line) was −1.454, and the Corrected *B* value (corrected for the mean locus length) was −3.441. The locus length of the first letter was 18.8 mm, and the mean locus length was 12.1 mm. (b) A sample case of PM in the copying task in patients with PD. In this case, the *B* value was −0.994, and the Corrected *B* value was −1.815. The locus length of the first letter was 18.5 mm, and the mean locus length was 13.9 mm. (c) The process of detecting PM. The scanned letters were skeletonized, and the locus length of each letter (the total number of pixels of skeletonized letter; 1 mm = 7.8788 pixels) was measured. To determine whether the written letters revealed PM, we conducted a linear regression analysis. The regression line was obtained by the least squares method (with the ordinal number of the letter as the dependent variable and the locus length of the letter as the independent variable), and the slope of the regression line (the *B* value) was calculated. The Corrected *B* value was also calculated because the absolute value of the *B* value increases in proportion to the mean locus length of the letters, which leads to overestimations of the decreases in the locus lengths in subjects with large letter sizes

We administered experimental tests for micrographia that consisted of the following components: freely writing and copying Japanese hiragana letters. In the PD patients, these writing tasks were performed while the patients were in the on‐medication state. For free writing, the subjects were required to write the Japanese hiragana letter “ma” repetitiously on a horizontal A4 (297 × 210 mm) sheet of paper in the vertical center of the sheet from left to right using a Pentel e‐sharp 0.5‐mm sharp pencil (Pentel Co., Ltd.; Figure 1a). For the copying task, as indicated in Figure 1b, seven sample letters of the Japanese hiragana letter “ma” were printed in black ink on a horizontal A4 sheet. The sample letters were printed in MS Mincho font style with a font size of 28 (area: 7.2 × 8.0 mm, locus length: 30.1 mm, interletter space: 2.4 mm), which was similar to the mean locus length that was derived from another group of 10 age‐ and sex‐matched healthy volunteers, from the middle of the left side. The subjects were required to write letters that matched the printed letters in size continuously using the same sharp pencil as that used in the free writing task. All subjects were instructed to write or copy letters at the same speed as they would in daily life and performed the free writing task first. They could see their handwriting during both tasks and the sample letters during the copying task. The time limit was 60 s for both tasks.

Each A4 sheet that was used in the two tasks was scanned at 200 dpi (2,340 × 1,654 pixels) with a 256‐level grayscale using an ApeosPort‐III C5500 (Fuji Xerox Co., Ltd.). These grayscale data were converted into binary data using Photoshop software (version 7.0; Adobe Inc.). The data for each letter on the sheets were skeletonized, and the locus length of each letter, which was defined by the total number of pixels of a skeletonized letter (1 mm = 7.8788 pixels), was considered the letter size and was measured using ImageJ software (http://rsb.info.nih.gov/ij; Figure 1c). We first used the criteria in Kim et al.'s previous study to determine whether the letters on each sheet revealed the presence of CM and/or PM, but none of the patients were found to have CM in both tasks, and two patients were found to have PM only in the copying task (Kim et al., 2005). Therefore, we decided to use our own criteria for detecting CM and PM. Our own criteria for detecting CM were as follows: (1) a locus length of the first letter smaller than the mean‐1.5*SD of the locus length of the first letter of the HCs; and (2) a mean locus length smaller than the mean‐1.5*SD of the mean locus length of the HCs. PM was considered present if the following criteria were met: (1) The regression line obtained by the least squares method (with the ordinal number of the letter set as the dependent variable and the locus length of the letter set as the independent variable) was statistically significant (*p* < .05); (2) the slope of the regression line (*B* value) was negative; and (3) the *B* value corrected for the mean locus length (Corrected *B* value) was less than the mean‐1.5*SD of the Corrected *B* values of the HCs (Figure 1c).

### 2.3 PET study

All forty patients with PD underwent a PET scan within the 2 weeks that preceded or followed the clinical assessments. Their regional cerebral metabolic rate of glucose (rCMRglc) utilization was measured using FDG‐PET. All patients fasted for more than 5 hr before the scan and were injected with 185–218 MBq FDG intravenously 1 hr prior to the scan. Thirty‐seven of the patients were prescribed antiparkinsonian drugs and were in the off‐medication state for more than 5 hr after taking the last dose. Three of the patients were de novo at the time of the PET scans and had not yet received antiparkinsonian drugs. Dynamic PET scans in the three‐dimensional mode were performed using a Siemens Biograph DUO PET scanner (Siemens Medical System, Inc.). The patients were scanned under a resting condition with their eyes closed and ears unplugged. To minimize the effects of external stimuli during the FDG uptake period of 1 hr, the patients remained in a quiet room wearing eye masks. The in‐plane and axial resolutions of the scanner were 3.38 and 3.88 mm, respectively. Attenuation correction was performed with a CT scan. The data obtained were reconstructed using ordered subset expectation maximization algorithms (16 subsets × 6 iterations) with a Gaussian filter with the following parameters: full width at half maximum = 2.0 mm, matrix size = 256 × 256, pixel size = 1.33 mm × 1.33 mm, and slice thickness = 2.0 mm. The PET images and the arterial input function measurements were used to determine the cerebral metabolic rate of glucose according to a model based on the autoradiographic technique. Unfortunately, we excluded two patients from the PET study because the quality of the PET images of these patients was insufficient for analysis.

The PET images were analyzed with statistical parametric mapping software version 12 (SPM12; Wellcome Centre of Imaging Neuroscience) implemented in MATLAB 9.1.0 (MathWorks Inc.). All PET images were normalized to the FDG template based on the Montreal Neurological Institute reference brain (resampled with a voxel size of 2 × 2 × 2 mm^3^). Then, all normalized images were smoothed using an isotropic Gaussian kernel of 10 mm to increase the signal‐to‐noise ratio and to compensate for differences in gyral anatomy across individuals. To reduce between‐subject variation in the global metabolic rates, we normalized the count of each voxel to the total count of the brain using proportional scaling.

### 2.4 Statistical analyses

The prevalence rates of CM and PM were compared between the two groups and between the two tasks using the chi‐square test. Two‐way repeated‐measures ANOVA was used to analyze the associations between the groups (the PD and HC groups) and the existence of a visual reference (the free writing and copying tasks) in the first letter size, mean letter size, and Corrected *B* value. In a post hoc analysis, Wilks' lambda was used to evaluate the simple main effects, and Bonferroni correction was used for multiple comparisons. These outcome measures were also compared between subjects only with CM (selective CM) and without CM and between subjects only with PM (selective PM) and without PM in each group by the Mann–Whitney *U* test. In addition, we investigated whether there were differences in the disease duration, daily levodopa equivalent dose (LED: regular levodopa dose 1 + slow‐release levodopa × 0.75 + bromocriptine × 10 + apomorphine × 10 + ropinirole × 20 + pergolide × 100 + pramipexole × 100 + [regular levodopa dose + (slow‐release levodopa × 0.75)] × 0.2 if taking entacapone), UPDRS motor scores, Brady/hypokinesia scores (the sum of the scores for finger taps, hand movements, rapid alternating movements of the hands, leg agility, and body brady/hypokinesia from the motor part of the UPDRS), and MMSE scores between the PD patients with and without CM or PM using the Mann–Whitney *U* test. Moreover, we evaluated whether there were associations between the mean letter size and Brady/hypokinesia score within patients with selective CM for each task and whether there were associations between the Corrected *B* value and Brady/hypokinesia score within patients with selective PM for each task using Spearman's rank correlation coefficient. Statistical analyses were performed using IBM SPSS statistics software (version 25.00; IBM SPSS Inc.), and *p* < .05 indicated statistical significance.

To determine which brain regions were most closely associated with the presence of CM or PM in patients, voxel‐based comparisons of rCMRglc between the patients with or without selective CM or PM and another group of 14 healthy controls (mean age, 64.0 ± 4.2 years; seven females and seven males; mean education attainment, 12.3 ± 2.5 years; mean MMSE score, 29.1 ± 1.3) who had not participated in experimental writing tasks and between the patients without selective CM or PM and the healthy controls who underwent only a PET scan were performed using two‐sample *t* tests in SPM12. Age and sex were included as nuisance variables. In addition, voxel‐based comparisons for rCMRglc between the PD patients with or without selective PM or CM were performed using two‐sample *t* tests. To confine our analysis to regions showing hypometabolism in the patients relative to the healthy controls, a resulting map of the comparison between the patients with selective CM or PM and the healthy controls, with a liberal statistical threshold (*p* < .05, uncorrected), for each task was used for masking (Abe et al., 2009). The patients' age, sex, LED, UPDRS motor part score, and MMSE score were included as nuisance variables.

Moreover, we conducted voxel‐based linear regression analysis for PM using glucose metabolic rate as the dependent variable and the Corrected *B* values for each task as the independent variable. The above masking was applied to restrict our analysis to regions showing hypometabolism in the patients relative to the healthy controls in each linear regression analysis. The patients' age, sex, LED, UPDRS motor part score, and MMSE score were included as nuisance variables in each model. In the linear regression analyses, patients with progressive macrographia were excluded. If progressive macrographia in patients is a pathological phenomenon, the brain regions detected in the analyses may be unreliable. Progressive macrographia was determined to be present if the following criteria were met: (1) the regression line (with the ordinal number of the letter as the dependent variable and the locus length of the letter as the independent variable) was statistically significant (*p* < .05); (2) the slope of the regression line (*B* value) was positive; and (3) the Corrected *B* value was greater than the mean+1.5*SD of the Corrected *B* values of the HCs.

The results were considered to exhibit statistically significant differences if the uncorrected *p*‐value was <.001. The minimum cluster size of each comparison or linear regression analysis was defined according to the expected cluster size, as computed by SPM 12.

## 3 RESULTS

### 3.1 Experimental writing tests

The prevalence rates of CM and PM in patients with PD and HC are shown in Table 2A. The prevalence of CM in free writing was 2.5% in the PD group and 0% in the HC group. The prevalence of PM in the free writing task was 15% in the PD group and 10% in the HC group. There were no significant differences in the prevalence of CM or PM between the groups in the free writing task. Alternatively, the prevalence of CM in the copying task was 2.5% in the PD group and 0% in the HC group. The prevalence of PM in the copying task was 17.5% in the PD group and 0% in the HC group. Although there was no significant difference in the prevalence of CM between the groups in the copying task, the prevalence of PM in the copying task was significantly higher in the PD group than in the HC group. Neither of the 2 (0%) patients with CM in either task had CM in both tasks, and three of the 10 (30%) patients with PM in either task had PM in both tasks. In addition, none of the 7 (0%) patients with either type of micrographia had both CM and PM in the free writing task, and one of the eight (12.5%) patients with either type of micrographia had both CM and PM in the copying task. There was no significant difference in the prevalence of CM or PM between the two tasks in the PD group (*p* = 1.000 for CM, *p* = .762 for PM) or in the HC group (*p* = 1.000 for CM, *p* = .147 for PM).

**TABLE 2:** The results of experimental writing test. (a) Parkinson's disease versus Healthy controls. (b) The subjects with versus without selective consistent or progressive micrographia

(a)				
Tasks	Variables	PD (*n* = 40)	HCs (*n* = 20)	*p*‐Value
Free writing	Prevalence of CM	1 (2.5%)	0 (0%)	.476
	Prevalence of PM	6 (15%)	2 (10%)	.591
	Prevalence of PMa	4 (10%)	2 (10%)	1.000
	First letter size	23.5 ± 6.9 mm	31.0 ± 13.2 mm	.025
	Mean letters size	23.2 ± 8.2 mm	28.7 ± 9.5 mm	.025
	Corrected *B* value	−0.24 ± 2.24	−0.04 ± 1.55	.733
Copying	Prevalence of CM	1 (2.5%)	0 (0%)	.476
	Prevalence of PM	7 (17.5%)	0 (0%)	.047
	Prevalence of PMa	3 (7.5%)	2 (10%)	.741
	First letter size	21.7 ± 3.7 mm	21.9 ± 4.8 mm	.817
	Mean letters size	22.8 ± 4.8 mm	25.3 ± 3.9 mm	.048
	Corrected *B* value	0.56 ± 2.07	1.10 ± 1.11	.276

(b)							
Subjects	Task	Variables	With CM	Without CM	With PM	Without PM	*p*‐Value
							PM
PD	Free writing	First letter size (mm)	9.8	21.6 (9.3, 26.8)	19.9 (10.1, 12.8)	21.7 (9.8, 31.9)	.324
		Mean letters size (mm)	14.4	22.0 (10.5, 34.0)	15.1 (8.9, 9.0)	22.5 (10.0, 31.7)	.005
		Corrected *B* value	0.13	0.07 (2.55, 10.64)	−3.43 (3.72, 5.40)	0.26 (2.28, 5.17)	<.001
		Age (years)	68.0	68.0 (13.0, 25.0)	65.0 (10.0, 14.0)	68.0 (13.0, 25.0)	.493
		Disease duration (years)	11.0	6.0 (6.0, 19.0)	6.0 (4.0, 6.0)	7.0 (6.0, 19.0)	.782
		UPDRS motor score	10.0	19.5 (11.0, 35.0)	20.0 (19.0, 28.0)	19.0 (11.0, 27.0)	.458
		Brady/hypokinesia score	3.0	7.0 (6.0, 13.0)	7.0 (6.0, 8.0)	7.0 (7.0, 13.0)	.900
		MMSE total score	30.0	28.0 (3.0, 6.0)	29.0 (3.0, 5.0)	28.0 (3.0, 6.0)	.324
		LED	700.8	450.0 (400.0, 1,250.0)	412.5 (262.5, 600.0)	522.5 (407.2, 1,250.0)	.425
	Copying	First letter size (mm)	14.6	21.6 (5.6, 15.9)	21.1 (4.2, 7.4)	22.0 (6.1, 16.6)	.288
		Mean letters size (mm)	13.6	21.9 (7.8, 20.1)	19.2 (5.7, 6.2)	22.4 (6.5, 20.4)	.001
		Corrected *B* value	−1.91	0.90 (2.62, 13.03)	−1.57 (1.52, 4.87)	1.04 (1.88, 8.83)	<.001
		Age (years)	76.0	68.0 (11.0, 25.0)	64.5 (11.0, 15.0)	68.0 (13.0, 25.0)	.470
		Disease duration (years)	7.0	6.0 (6.0, 19.0)	6.0 (4.0, 9.0)	7.0 (6.0, 19.0)	.566
		UPDRS motor score	11.0	19.5 (11.0, 35.0)	21.5 (10.0, 17.0)	18.5 (11.0, 30.0)	.776
		Brady/hypokinesia score	5.0	7.0 (7.0, 13.0)	8.0 (6.0, 11.0)	6.0 (7.0, 11.0)	.245
		MMSE total score	26.0	28.0 (4.0, 6.0)	29.0 (3.0, 5.0)	28.0 (3.0, 6.0)	.324
		LED (mg/day)	545.0	450.0 (400.8, 1,250.0)	435.0 (425.0, 575.0)	500.0 (407.2, 1,250.0)	.671
HCs	Free writing	First letter size (mm)	ND	29.8 (16.7, 54.1)	59.4 (ND, 19.3)	27.0 (14.4, 30.2)	.011
		Mean letters size (mm)	ND	28.2 (11.5, 38.6)	45.7 (ND, 11.4)	26.9 (11.4, 30.7)	.021
		Corrected *B* value	ND	0.02 (1.46, 6.17)	−3.45 (ND, 0.71)	0.17 (1.47, 369)	.011
		Age	ND	69.0 (9.0, 16.0)	64.0 (ND, 65.0)	70.0 (8.0, 16.0)	.126

Note

Data are given as mean ± *SD* in Table 2A and as median (quartile, range) in Table 2B. The brady/hypokinesia score = the sum of scores finger taps, hand movements, rapid alternating movements of hands, leg agility, and body brady/hypokinesia from UPDRS motor part. LED = regular levodopa dose 1 + slow‐release levodopa × 0.75 + bromocriptine × 10 + apomorphine × 10 + ropinirole × 20 + pergolide × 100 + pramipexole × 100 + [regular levodopa dose + (slow‐release levodopa × 0.75)] × 0.2 if taking entacapone.

The two‐sample *t* test with the Bonferroni correction (a post hoc analysis after a two‐way repeated‐measures ANOVA) was used except for prevalence (the chi‐square test) in Table 2A, and the Mann–Whitney *U* test was used in Table 2B.

Abbreviations: CM, consistent micrographia; HCs, healthy controls; LED, levodopa equivalent dose; ND, not detected; PD, Parkinson's disease; PM, progressive micrographia; PMa, progressive macrographia; UPDRS, Unified Parkinson's Disease Rating Scale.

**FIGURE 2:**
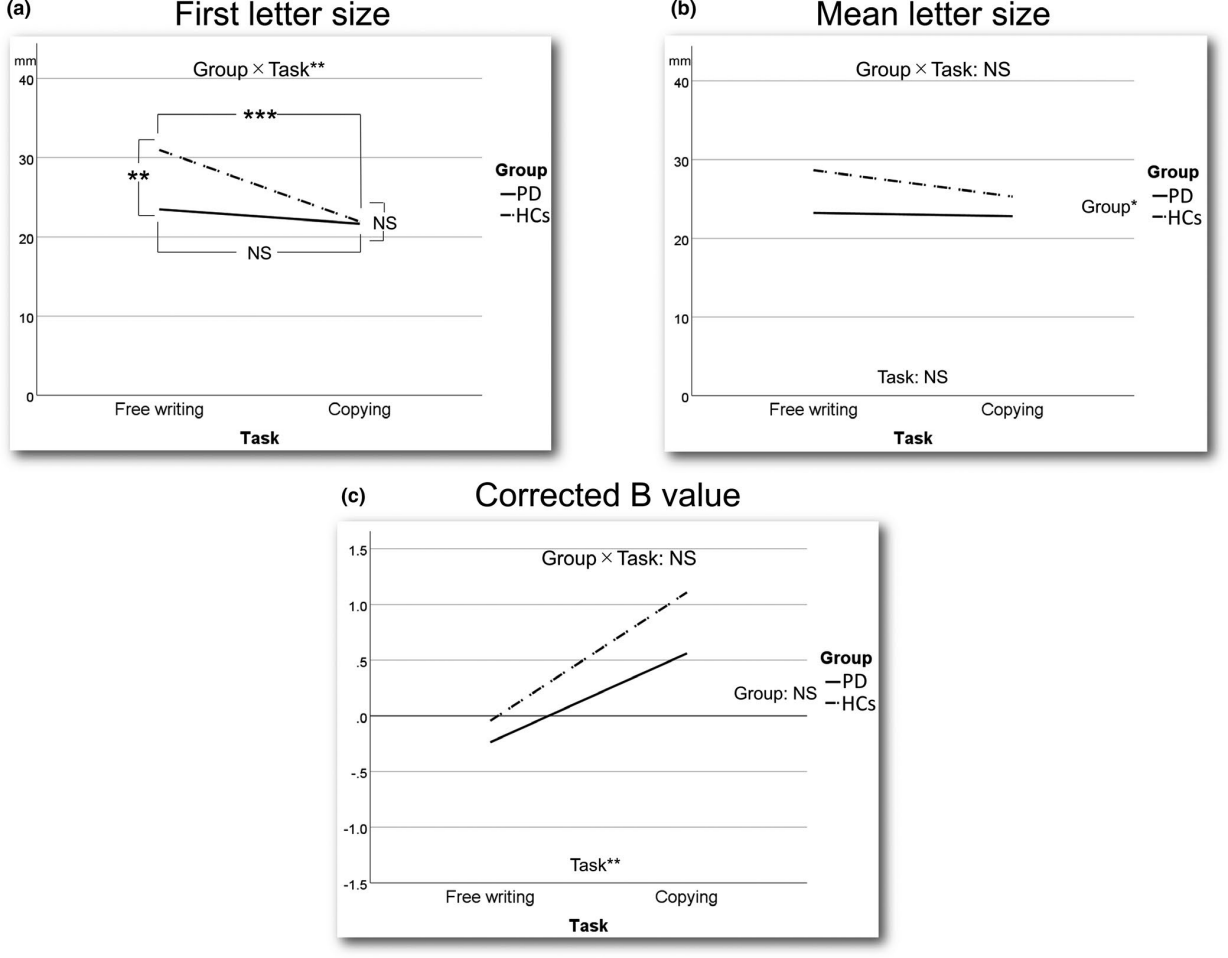
The results of two‐way repeated‐measures ANOVA. (a) For the size of the first letter, there was a significant interaction between Group (Parkinson's disease and healthy controls) and Task (free writing and copying). (b) For the mean letter size, there was no significant interaction between Group and Task and no significant main effect of Task, whereas there was a significant main effect of Group. (c) For the Corrected *B* value, there were no significant interactions between Group and Task and no significant main effect of Group, whereas there was a significant main effect of Task. *p < .05. **p < .005. ***p < .001. HCs, healthy controls, NS, not significant; PD, Parkinson's disease

The size of the first letter, mean letter size, and Corrected *B* value for each task in each group are shown in Table 2A and Figure 2. First, there were significant main effects of Group [*F* (1, 58) = 6.082, *p* = .017] and Task [*F* (1, 58) = 19.826, *p* < .001] and a significant interaction between Group and Task [*F* (1, 58) = 8.782, *p* = .004] for the first letter size according to the two‐way repeated‐measures ANOVA results (Figure 2a). The simple main effect tests showed that the mean first letter size in the free writing task in the PD group was significantly smaller than that in the HC group, whereas there was no significant difference in the mean first letter size in the copying task between the two groups. In addition, there was no significant difference in the mean first letter size in the PD group between the two tasks (Wilks' lambda = 0.972, *F* (1, 58) = 1.663, *p* = .202), whereas the mean first letter size in the copying task was significantly smaller than that in the free writing task in the HC group (Wilks' lambda = 0.738, *F* (1, 58) = 20.624, *p* < .001). Second, there was a significant main effect of Group [*F* (1, 58) = 6.063, *p* = .017] only and no significant main effect of Task [*F* (1,58) = 3.669, *p* = .060] or interaction effect between Group and Task [*F* (1,58) = 2.203, *p* = .143] for the mean letter size (Figure 2b). Last, there was a significant main effect of Task [*F* (1, 58) = 12.824, *p* = .001] only and no significant main effect of Group [*F* (1, 58) = 0.663, *p* = .419] or interaction effect between Group and Task [*F* (1, 58) = 0.426, *p* = .516] for the Corrected *B* value (Figure 2c).

The first letter size, mean letter size, and Corrected *B* value in the subjects with and without selective CM or PM in each task and the disease duration, UPDRS motor score, brady/hypokinesia score, MMSE total score, and LED in the PD patients with and without CM or PM in each task are shown in Table 2B. We could not compare these measures in each task between the patients with and without selective CM and could not evaluate whether there was an association between the mean letter size and brady/hypokinesia score within patients with selective CM in each task because there was only one patient with selective CM in each task. Although the mean letter size of the PD patients with selective PM was significantly smaller than that of the patients without selective PM in both tasks, there was no significant difference in the mean first letter size between PD patients with and without selective PM in both tasks. In contrast, the mean first letter size and mean letter size of the HCs with selective PM were significantly larger than those of the HCs without selective PM in the free writing task (Figure S1). There was no significant difference in the mean disease duration, UPDRS motor and Brady/hypokinesia scores, MMSE total score, or LED between PD patients with and without selective PM in both tasks. There were no correlations between the Corrected *B* value and Brady/hypokinesia score in both tasks in the PD patients with selective PM.

### 3.2 PET study

We could not perform voxel‐based comparisons of glucose metabolic rate between the patients with or without selective CM and the healthy controls, who had not participated in the writing tests, because there was only one patient with selective CM in each task.

**FIGURE 3:**
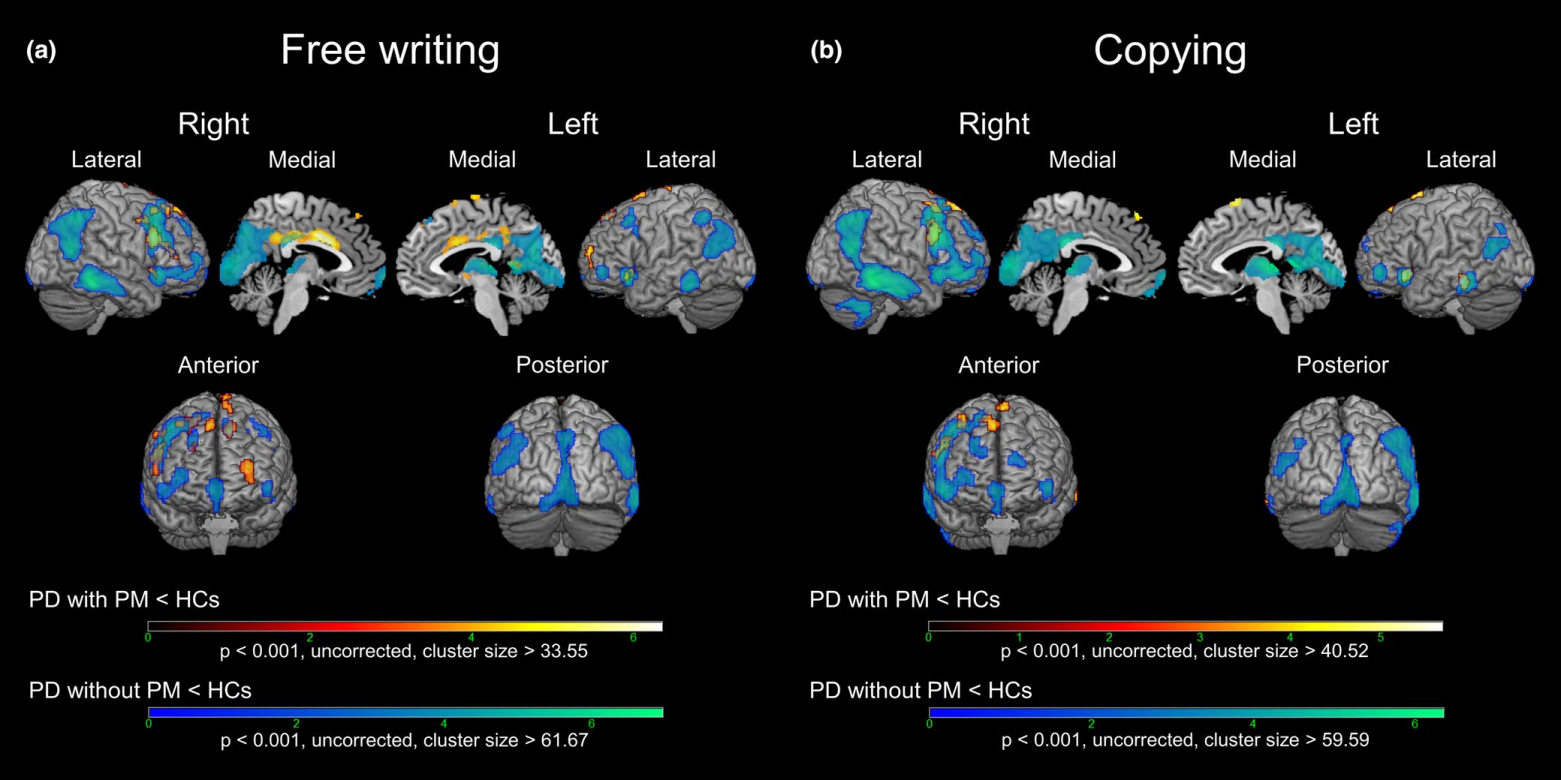
The results of voxel‐based comparisons of brain glucose metabolism between patients with Parkinson's disease (PD) and healthy controls (HCs). (a) PD patients with progressive micrographia (PM) versus HCs (black‐red‐yellow‐white) and PD patients without PM versus HCs (blue‐green) in the free writing task. (b) PD patients with progressive micrographia (PM) versus HCs (black‐red‐yellow‐white) and PD patients without PM versus HCs (blue‐green) in the copying task. The models were adjusted for the possible confounding effects of age and sex. The colored bars indicate the t values. HCs, healthy controls; PD, Parkinson's disease; PM, progressive micrographia

The results of the voxel‐based comparisons of glucose metabolic rate between the patients with or without selective PM and the healthy controls for each task are shown in Figure 3 and Table S1. The patients with selective PM in the free writing task showed clusters of hypometabolism in the left and right anterior and middle cingulate cortexes (ACC and MCC), left SMA (pre‐SMA and rostral SMA‐proper), and left and right superior frontal gyrus (SFG) compared with the healthy controls, which was not observed in the comparison between the patients without selective PM and the healthy controls. Alternatively, the patients with selective PM in the copying task specifically showed clusters of hypometabolism in the left pre‐SMA and right SFG. There was no significant cluster of hypermetabolism in the patient with selective PM in both tasks compared with the healthy controls.

**FIGURE 4:**
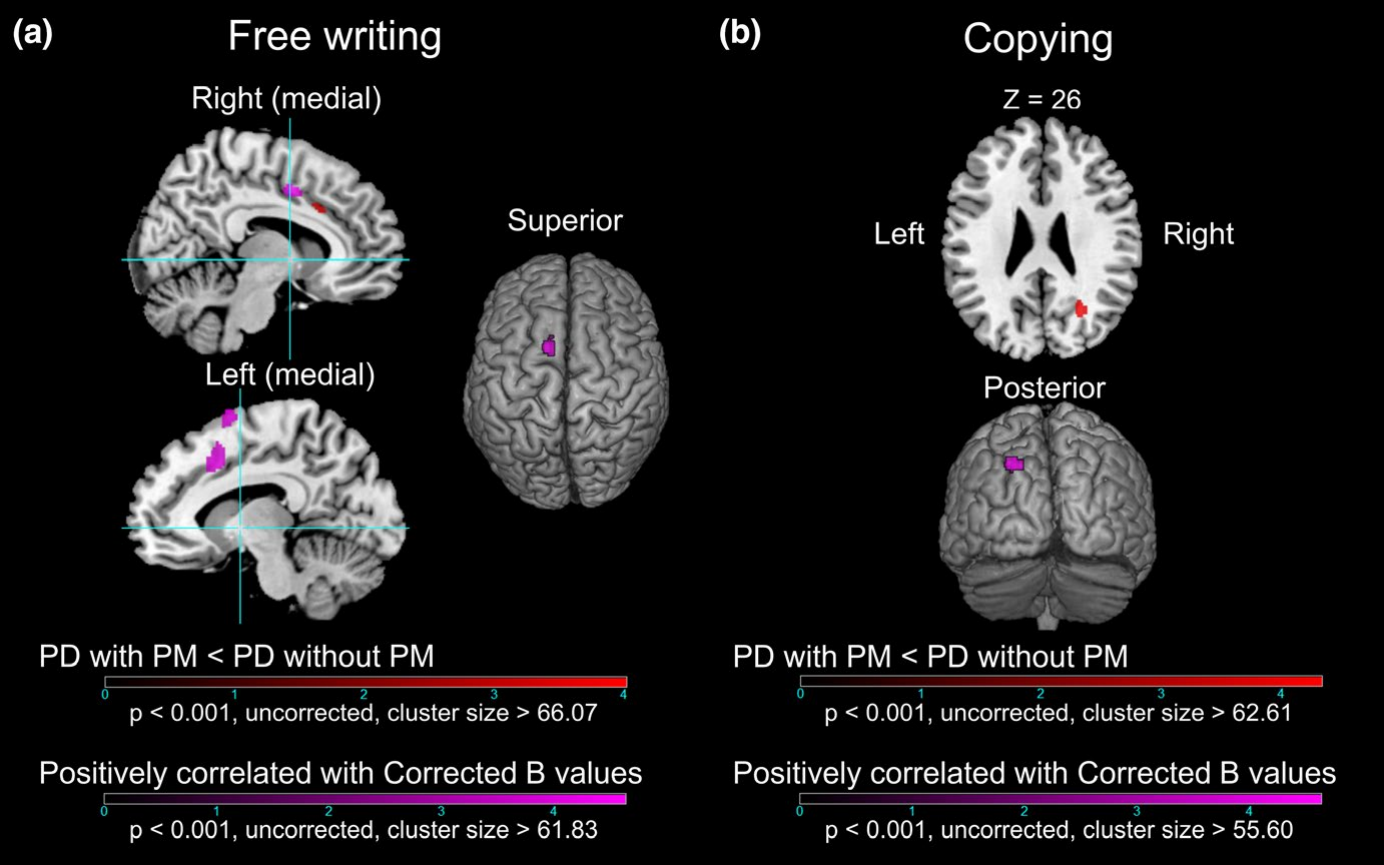
The results of the voxel‐based comparisons of brain glucose metabolism between Parkinson's disease (PD) patients with and without progressive micrographia (PM) and voxel‐based linear regression analyses within PD patients. (a) The PD patients with versus without PM (black‐red) and the clusters in which glucose metabolism was positively correlated with the Corrected *B* values (black‐violet) in the free writing task. The blue vertical line in the sagittal plane penetrates the anterior commissure and is perpendicular to the anterior–posterior commissure line (sky blue horizontal line), which was used to divide the supplementary motor area (SMA) into the pre‐SMA (anterior part) and SMA‐proper (posterior part). (b) The PD patients with versus without PM (black‐red) and the clusters in which glucose metabolism was positively correlated with the Corrected *B* values (black‐violet) in the copying task. To confine our analysis to regions showing hypometabolism in the patients relative to the healthy controls, a resulting map of the comparison between the patients with CM and selective PM and the healthy controls, with a liberal statistical threshold (*p* < .05, uncorrected), for each task was used for masking. The colored bars indicate the t values. PD, Parkinson's disease; PM, progressive micrographia

The results of the comparisons of glucose metabolic rate between the patients with and without selective PM are shown in Figure 4 and Table 3. The patients with selective PM in the free writing task showed a cluster of hypometabolism in the right MCC compared with the patients without selective PM. In contrast, the patients with selective PM in the copying task showed a cluster of hypometabolism in the right superior occipital gyrus (SOG). There was no cluster of hypermetabolism in the patient with selective PM in both tasks compared with the patients without selective PM.

**TABLE 3:** The results of FDG‐PET study. (a) Brain regions in which regional cerebral glucose metabolism was decreased in PD patients with progressive micrographia compared to those without progressive micrographia. (b) Brain regions in which regional cerebral glucose metabolism was positively correlated with corrected *B* values in PD patients without progressive micrographia

(a)						
Task	Regions (BA)	MNI coordinates			*t*‐value	Cluster size
		*x*	*y*	*z*		
Free writing	Right middle cingulate cortex (32)	10	20	32	3.88	67
Copying	Right superior occipital gyrus (19)	26	−66	24	4.33	104

(b)						
Task	Regions (BA)	MNI coordinates			*t*‐value	Cluster size
		*x*	*y*	*z*		
Free writing	Left supplementary motor area (6)	−12	6	70	4.64	71
	Right middle cingulate cortex (24)	6	2	42	4.53	282
	Left middle cingulate cortex (24)	−4	2	42		
	Left middle cingulate cortex (32)	−8	14	42		
Copying	Left superior occipital gyrus (19)	−26	−88	44	4.61	58

Abbreviations: BA, Brodmann area; MNI, Montreal Neurological Institute.

The results of the voxel‐based linear regression analyses on each task in the PD group are also shown in Figure 4 and Table 3. There were four patients with progressive macrographia in the free writing task and three patients with progressive macrographia in the copying task. Therefore, these patients were excluded from the linear regression analysis. The Corrected *B* value for the free writing task was significantly positively correlated with the glucose metabolic rate of the clusters in the left pre‐SMA and the left and right MCC. Additionally, the Corrected *B* value in the copying writing task was significantly positively correlated with the glucose metabolic rate of the cluster in the left SOG. There were no significant negatively correlated regions in either task.

## 4 DISCUSSION

### 4.1 PET study

In the present study, the PD patients with selective PM in the free writing task showed glucose hypometabolism in the left and right ACC and MCC, left pre‐SMA and SMA‐proper, and left and right SFG compared with the healthy controls, which was not observed in the comparison between the patients without selective PM and the healthy controls. In particular, glucose hypometabolism present in the anterior part of the right MCC (Brodmann area 32) was also observed in patients with selective PM compared with those without selective PM. The anterior part of the MCC was thought to include the CMAr, as shown in the macaque monkey (Matelli, Luppino, & Rizzolatti, 1991). The CMAr is known to be associated with the monitoring of preresponse conflicts and response errors (Ridderinkhof, Ullsperger, Crone, & Nieuwenhuis, 2004). Previous primate studies have demonstrated that the CMAr receives inputs from various areas, including the parietal association cortex, temporal association cortex, occipital cortex, prefrontal cortex, pre‐SMA, premotor area, amygdala, parahippocampal cortex, insula cortex, and thalamus (Morecraft & Van Hoesen, 1998; Vogt & Pandya, 1987). Therefore, it is possible that the CMAr plays a crucial role in monitoring whether actual handwriting movements are desirable for maintaining letter size. Our results demonstrated a right‐hemispheric dominance of the CMAr related to PM in free writing in PD patients. In a previous functional MRI study of illusory palmar flexion that was elicited by tendon vibration, the right premotor cortex, right inferior parietal lobule including high‐somatosensory area, and right superior temporal gyrus exhibited significantly higher activations than the corresponding left regions when the subjects experienced an illusory movement of the left or right hand, which demonstrated a right‐hemispheric dominance in the perception of limb movement (Naito et al., 2005). The right CMAr might be more advantageous than the left CMAr in monitoring actual handwriting movements because there is a large amount of information about upper limb kinesthesia. In addition, a previous primate study revealed that the CMAr is activated more by self‐paced forelimb movements than by sensorially triggered forelimb movements (Shima et al., 1991). The free writing task performed in the current study required the subjects to continue self‐paced letter writing. Therefore, dysfunction of the right CMAr might lead to PM in PD patients in the free writing task. Glucose hypometabolism evident in the left CMAr (Brodmann area 32) and the right MCC (Brodmann area 24) was associated with a decrease in letter size in the PD patients. This area with glucose hypometabolism in the right MCC is also thought to be a part of the CMAr. It is suggested that a large decrease in letter size during free handwriting arises from dysfunction in the bilateral CMAr in PD patients.

Glucose hypometabolism in the left pre‐SMA and SMA‐proper was also observed only in the patients with selective PM in the free writing task. Although the glucose metabolic rate in the left pre‐SMA in the patients with selective PM was not significantly lower than that in the patients without selective PM, the presence of glucose hypometabolism in this area was significantly associated with smaller letter sizes in the PD patients. Previous primate studies have demonstrated that the pre‐SMA plays a key role in the preparation, procedural learning, and planning of complex voluntary movements (Matsuzaka, Aizawa, & Tanji, 1992; Roland, Larsen, Lassen, & Skinhøj, 1980). These functions are crucial for maintaining letter sizes in handwriting tasks. The activation of the pre‐SMA and/or SMA‐proper during handwriting tasks has been reported in several studies (Matsuo et al., 2000; Nackaerts, Michely, et al., 2018; Sosnik, Flash, Sterkin, Hauptmann, & Karni, 2014; Wu et al., 2016). In addition, dysfunction of the pre‐SMA and/or SMA‐proper in PD patients during handwriting tasks has been frequently indicated in previous studies (Nackaerts, Michely, et al., 2018; Nackaerts, Nieuwboer, et al., 2018; Wu et al., 2016). Wu et al. reported that the activities of the left pre‐SMA, right CMAr, and left posterior putamen were correlated with decreases in letter sizes in PD patients with selective PM (Wu et al., 2016). These findings, except for those of the left posterior putamen, were consistent with our results. Previous primate studies have shown that the pre‐SMA is strongly associated with the CMAr (Hoffstaedter et al., 2014; Takada et al., 2001). The relation between these areas is thought to be extremely important in planning and exhibiting desirable handwriting movements and maintaining the size of letters. Thus, dysfunction of the pre‐SMA and CMAr might cause difficulty in modifying handwriting movements that are inadequate for maintaining the size of letters, leading to the emergence of PM in free writing tasks in PD patients.

We could not detect lesions in the putamen that were related to PM in the free writing task in the PD patients. Many case studies and several functional MRI studies on micrographia have focused on the involvement of the putamen, which is linked to dysfunction of the basal ganglia motor circuit (Ishihara et al., 2006; Lewitt, 1983; Ling et al., 2012; Münchau et al., 2000; Wagle Shukla et al., 2012; Yoshida et al., 1989). One possible reason is that dopaminergic medication affects the metabolism of the brain. Previous studies have reported that the dopaminergic medication status has a significant impact on the glucose metabolism of the brain. In particular, the CMRglc values in subcortical structures such as the striatum and thalamus have been shown to increase by the administration of dopaminergic medication (Berding et al., 2001; Feigin et al., 2001). Although we withheld dopaminergic medication for the 5 hr immediately preceding the PET scan for each patient, this washout duration is shorter than the washout duration that has been used in previous studies. Therefore, we might have failed to detect striatal metabolic abnormalities due to the effects of residual dopaminergic agents. Another possible reason is that the damage to the neocortex caused by a Lewy body pathology affects the appearance of PM in free handwriting in PD patients more than dysfunction of the basal ganglia. Although dysfunction of the basal ganglia motor circuit is a key factor in patients presenting major symptoms such as bradykinesia, tremor, and gait abnormalities, the prevalence rates of PM in PD patients in this and several previous studies were not so high (Kim et al., 2005; McLennan et al., 1972). In addition, levodopa reportedly did not lead to improvements in PM in PD patients (Wu et al., 2016). Moreover, PM sometimes manifests as the initial symptom of PD (McLennan et al., 1972). Therefore, we speculated that dysfunction of the CMAr and pre‐SMA due to a Lewy body pathology is essential for the emergence of PM in free writing in PD patients.

Additionally, the PD patients with selective PM in the copying task showed glucose hypometabolism in the left pre‐SMA and right SFG compared with the healthy controls, which was not observed in the comparison between the patients without selective PM and the healthy controls. On the other hand, glucose hypometabolism was observed in the right SOG near the parietooccipital sulcus and the cuneus (the rostral part of Brodmann area 19) in the patients with selective PM compared with those without selective PM. Moreover, although the glucose metabolic rate of the left SOG near the parietooccipital sulcus and the cuneus (the caudal part of Brodmann area 19) in the patients with selective PM in the copying task was not significantly lower than that in the patients without selective PM, the presence of glucose hypometabolism in this area was significantly associated with smaller letter sizes in the PD patients. These regions are associated with visual function and include the lateral areas of V3A (hOc4d), according to the studies by Catani and Thiebaut de Schotten (2012) and Malikovic et al. (2016). These areas are considered part of the dorsal stream of visual processing, which plays a significant role in sensorimotor transformations for visually guided actions (Goodale & Milner, 1992). Previous functional MRI studies have suggested that the functions of the V2 and V3 areas are to provide coherent and global motion processing (Braddick et al., 2001). In addition, visual motion learning in the visual hemifield increased the activity of the V2 and V3A areas contralateral to the trained visual hemifield (Furlan & Smith, 2016). A previous study on the kinematic analysis of upper limb trajectories reported that PD patients continuously rely on visual information for ongoing movement correction, and the absence of visual feedback aggravates the symptoms of bradykinesia (Flash, Inzelberg, Schechtman, & Korczyn, 1992). Therefore, we suggest that PD patients with PM in copying tasks are unable to adequately use visual information on their own handwriting and hand motions as cues for maintaining the size of letters. Nackaerts et al. found that visual cueing during handwriting tasks increases functional connectivity from the left MT/V5 area, SMA, and premotor area to the left SPL in both patients with PD and healthy controls (Nackaerts, Michely, et al., 2018). It is possible that impairments in visual motion perception due to dysfunction of the V3A areas and SMA are compensated by the MT/V5 area. As with dysfunction of the CMAr in the PD patients with PM in free writing, our results demonstrated a right‐hemispheric dominance of the SOG related to PM in PD patients in the copying task. Many previous case studies have demonstrated the superiority of the right hemisphere for spatial processing, represented by the appearance of left unilateral spatial neglect due to right hemisphere injuries (Corbetta & Shulman, 2011; Hillis et al., 2005). In PD patients, global visuospatial attention might be crucial for acquiring visual references that are used for maintaining the size of letters in handwriting against brady/hypokinesia. Furthermore, it is suggested that the presence of dysfunction of the bilateral V3A can increase the rate at which the size of letter decrease in copying tasks in PD patients with PM.

### 4.2 Experimental writing tests

We could not detect CM either on the free writing or coping task by using Kim et al.'s criteria (Kim et al., 2005). One possible reason is that one of characteristics of Japanese letters is called “harai (sweeping).” Kim et al. defined letter size as the area of a rectangle outlined by the upper, lower, left, and right margins of each letter. However, if there was a long “harai” in a written letter, the area of the rectangle became much larger, while the locus length of the letter became slightly longer (Figure S2). In the present study, the prevalence of CM in the free writing or copying task was eventually found to be only 2.5% in the PD patients, even though the cutoff value of the first and mean letter sizes were set to be the mean −1.5 *SD* of those of the HCs. Criteria for CM are very difficult to define. One reason is that there are individual differences in handwriting sizes. Another is that it is hard to confirm whether the handwriting sizes of participants decrease before PD onset. A previous study reported that the prevalence of CM or PM in signatures was 15%, which was defined by a longitudinal, but not quantitative, analysis (McLennan et al., 1972). The prevalence in the present study was considerably lower than that reported in the previous study. Therefore, unfortunately, we could not detect the regions with abnormal glucose metabolic rates that were associated with the severity of CM in the PD patients. Furthermore, both large‐scale and longitudinal studies are needed to confirm the criteria for CM.

The prevalence of PM in PD patients in free writing was 15% in the present study. We decided to use our own criteria for PM because we could not detect PM in the free writing by using Kim et al.'s criteria, even though there were PD patients with PM whose *B* value (by Kim et al.'s definition) was greater than the mean −2 SD of that of the HCs (Figure 1; Kim et al., 2005). The prevalence of PM in free writing in the current study was naturally higher than that in Kim et al.'s study, almost the same as that in McLennan et al.'s study, but lower than those in Shukla et al.'s (51.5%) and Zham et al.'s (66%) studies (Kim et al., 2005; McLennan et al., 1972; Wagle Shukla et al., 2012; Zham et al., 2019). In Shukla et al.'s and Zham et al.'s studies, the authors used the English letters, and the subjects were required to write a single letter twenty times on a paper. The areas (height * width) of the first five trials and of the last five trials were measured, and PM was defined as a decrease in the area by ≥30% (Shunkla et al.'s study) or ≥10% (Zham et al.'s study). Although their criteria for PM placed great importance on the decrement of letter sizes in the last phase, Kim et al.'s study and our study mainly focused on the sequence effect of handwriting among all trials, which might lead to the lower prevalence of PM in Kim et al.'s and our studies. Nonetheless, a uniform, validated method for evaluating PM is needed in future research.

In the current study, the prevalence of PM in the free writing task was 10% in the HC group, and there was no significant difference in the Corrected *B* values or the prevalence of PM in the free writing task between the PD and HC groups. In addition, if we did not set the cutoff value of the corrected *B* value at the mean −1.5 *SD* of that of the HCs, the prevalence of PM in the free writing task would have been 25% in the PD group and 20% in the HC group. These findings indicate that some elderly people might have PM in free handwriting. However, the first letter and the mean letter sizes of HCs with selective PM were significantly larger than those of the HCs without selective PM. The mechanism of PM in the HCs might be different from that in the PD patients because PM in the HCs can be caused by adjusting the size of the first letters, which were unexpectedly larger than the size of the other letters. However, we cannot unconditionally deny that PM in both PD patients and HCs might share the same mechanism. To the best of our knowledge, no studies have systemically investigated the prevalence of CM or PM in elderly people. Moreover, previous studies in PD patients with micrographia did not report the prevalence in healthy controls except for Zham et al.'s study (Kim et al., 2005; McLennan et al., 1972; Wagle Shukla et al., 2012; Zham et al., 2019). In Zham et al.'s study, the prevalence of PM was 16.7% in free writing. However, the mean size of first letter was not reported in the study. Additional studies are needed to clarify this issue.

In the two‐way repeated‐measures ANOVA results, the first letter and mean letter sizes with visual references in the PD patients were not significantly different than those in the HCs. This finding was previously observed in a study by Nackaerts, Michely, et al. (2018). The prevalence of CM in the copying task was slightly higher than that in the free writing task in the PD patients, although this difference was not statistically significant. This trend was also reported by Kim et al. (2005). However, in the current study, the reason was not that the difference in the mean letter size between the PD patients and HCs in the copying task became larger than that in free writing but rather that the *SD* of the mean letter size in the copying task became smaller than that in free writing in the HCs. Therefore, CM was detected more accurately in the copying task in the PD patients than in the HCs because the effect of visual cueing on the sizes of the written letters was stronger in the HCs. In contrast, the Corrected *B* value in the copying task was larger than that in free writing in the PD patients. This trend was contradictory to the results of Kim et al.'s study (Kim et al., 2005). Our results indicate that a visual reference can aid in preventing the size of letters from decreasing gradually in PD patients. Therefore, tasks involving copying letters may not always be superior to free writing tasks in detecting PM in PD patients. However, we could not simply compare the results of these studies. One possible reason is that there were differences in the copying tasks. Although we used only Japanese Kana letters for the copying task, Kim et al. used not only Korean letters but also Luria loops (Kim et al., 2005). The prevalence of PM in copying Luria loops was slightly higher than that in copying letters in the aforementioned study. Thus, figure copying tasks might be more effective than letter copying tasks in detecting PM in PD patients. However, not all of the PD patients with PM in free writing exhibited PM in copying. Furthermore, the lesions with glucose hypometabolism associated with PM in the free writing task were different from those associated with PM in the copying task in the current study. It was strongly suggested that there are different occurrence mechanisms for PM between free writing and copying tasks in PD patients.

Although we could not reliably detect CM, the first letter and mean letter sizes in free writing in the PD patients with selective PM were significantly smaller than those without selective PM, and the Corrected *B* values in the PD patients with selective CM were also smaller than those without CM. These results indicate that some patients with selective PM in the current study actually had CM in free writing. According to Wu et al., most PD patients with micrographia present both CM and PM, which is inconsistent with our results (12.5%) (Wu et al., 2016). Longitudinal studies need to be conducted to confirm the criteria for CM and resolve the inconsistency in results. Regardless of the type of micrographia an individual presented or task he or she performed, brady/hypokinesia was not more severe in the PD patients with micrographia than in those without micrographia, and the mean letter sizes or Corrected *B* values in the PD patients with selective CM or PM were not significantly correlated with the severity of brady/hypokinesia in the current study. These findings indicate that the severity of micrographia in PD patients may be independent of the severity of brady/hypokinesia. However, previous studies have demonstrated that the motor or bradykinesia scores in PD patients with micrographia were significantly correlated with the severity of micrographia (Kim et al., 2005; Wagle Shukla et al., 2012). In addition, dysfunction of the pre‐SMA and/or SMA‐proper and striatum observed in previous studies and the current study in the free writing task in PD patients is thought to also be related to the severity of brady/hypokinesia in PD patients (Turner, Grafton, McIntosh, Delong, & Hoffman, 2003). Therefore, it is unreasonable that the mechanism of micrographia is wholly independent of that of brady/hypokinesia in PD patients. In the current FDG‐PET study, we found that the appearance of PM was associated with dysfunction of the CMAr in free writing and with dysfunction of the SOG in copying. It is thought that the CMAr cues the pre‐SMA and SMA‐proper in response to the somatosensory and visuospatial information derived from the parietal association area and occipital cortex during automatic and self‐paced movements (Morecraft & Van Hoesen, 1998; Shima et al., 1991; Vogt & Pandya, 1987; Wu et al., 2016). On the other hand, information from the SOG is thought to be important for the online control of visually guided movements, which is also associated with the function of the PMC (Furlan & Smith, 2016; Hoshi & Tanji, 2000). We speculated that if the functions of the CMAr, SOG, and their related areas are maintained, the brady/hypokinesia symptoms might be more easily attenuated in writing letters of the same size by hand than other movements such as walking or standing. This characteristic of handwriting might cloud the relationship between micrographia and brady/hypokinesia.

### 4.3 Limitations

The current study has several limitations. One limitation is that we used different healthy controls in the experimental writing tests and PET study. It is preferable to investigate the glucose metabolism of brain regions that show significant interactions between the disease and the presence of micrographia by using data from experimental writing tests and FDT‐PET. However, we could not perform the experimental writing test in healthy controls in whom FDG‐PET scans were performed. Another limitation is that we did not examine whether PD patients with PM in the copying task had impaired visual motion perception. Additional studies are needed to clarify the relationship between PM in copying in PD patients and visual motion dysfunction.

Moreover, some PD patients had progressive macrographia in the present study. Our results and those of Kim et al.'s study revealed that macrographia can also occur in PD patients (Kim et al., 2005). However, the prevalence of progressive macrographia in PD patients was not found to be significantly different from that in HCs in either task in the current study. The results might be related to the size of the reference letter. The locus length of the sample letters used as references was very similar to the mean locus length that was derived from another group of 10 healthy volunteers. However, the mean size of the first letters of the HCs in the copying task was smaller than that in the free writing task and was nearly the same as that of the PD patients in the copying task. Potentially, the size of the letters in the copying task gradually increased because the size of the reference letter was smaller than that of participants' usual letters in free writing. Therefore, the type of macrographia we detected might not be a pathological phenomenon. Additional studies are needed to clarify this issue.

## 5 CONCLUSIONS

Our findings revealed that PM is independent of other motor symptoms and is related to dysfunction of the right CMAr, which leads to difficulty in monitoring whether actual handwriting movements are desirable for maintaining the size of letters during automatic and self‐paced writing by hand. In contrast, our findings clarified that PM in copying is associated with dysfunction of the right SOG, which causes a lack of visual information about an individual's handwriting and hand motions. Thus, we concluded that the appearance of PM in PD patients differs depending on the regional differences in dysfunction in motor and visuospatial areas. In the future, the occurrence mechanism of micrographia may be clarified by the development of a uniform, validated method for evaluating micrographia and studying the type of damage to the neocortex that has a direct or an indirect connection to the corticobasal ganglia networks.

## CONFLICT OF INTEREST

None declared.

## AUTHOR CONTRIBUTIONS

SK, YN, TB, AT, HF, and EM involved in the study design. SK, MS, KK, YG, MU, YH, and AT performed the data acquisition and analysis. SK wrote the manuscript. KS edited the manuscript.

## Supporting information

Supplementary Material

## Data Availability

The data that support the findings of this study are available from the corresponding author upon reasonable request.
