# Supplementary material for: Neural substrates underlying progressive micrographia in Parkinson's disease
Source: Brain Behav. 2020 Jun 18;10(8):e01669. doi: 10.1002/brb3.1669 (PMC7428504; doi:10.1002/brb3.1669)
Supplement: Supplementary file 1 — Supplementary Material [file BRB3-10-e01669-s001.docx]

**Supplementary Table 1. The results of FDG-PET study.**

A. Brain regions in which regional cerebral glucose metabolism was decreased in PD patients with progressive micrographia compared to healthy controls

| Task | Regions (BA) | MNI coordinates | | | t-value | Cluster size |
| --- | --- | --- | --- | --- | --- | --- |
|  |  | x | y | z |  |  |
| Free writing | Right anterior cingulate cortex (24)  Right mid-cingulate cortex (24)  Right mid-cingulate cortex (23) | 8  4  8 | 14  2  -44 | 28  34  34 | 6.35  6.23  5.82 | 1534 |
|  | Left thalamus  Left thalamus  Left thalamus | -18  -10  -10 | -30  -20  -2 | 8  10  4 | 6.09  5.09  4.51 | 441 |
|  | Right thalamus  Right thalamus  Right thalamus | 14  12  14 | -10  -22  -20 | 16  -2  10 | 5.98  5.74  5.33 | 636 |
|  | Right inferior frontal operculum (44)  Right precentral gyrus (6)  Right inferior frontal triangularis (45) | 54  58  58 | 18  4  40 | 28  44  10 | 5.76  4.39  4.23 | 322 |
|  | Left superior frontal gyrus (9) | -8 | 44 | 48 | 5.34 | 124 |
|  | Left superior frontal gyrus (10) | -28 | 62 | 16 | 5.18 | 123 |
|  | Right superior frontal gyrus (9) | 8 | 42 | 54 | 5.12 | 67 |
|  | Right middle frontal gyrus (9)  Right middle frontal gyrus (9)  Right superior frontal gyrus (8) | 24  22  22 | 34  46  32 | 42  32  56 | 5.12  4.90  4.45 | 180 |
|  | Left inferior orbitofrontal gyrus (47) | -42 | 20 | -6 | 4.79 | 103 |
|  | Left precuneus (30) | -4 | -52 | 10 | 4.73 | 40 |
|  | Left supplementary motor area (6) | -6 | -11 | 78 | 4.66 | 64 |
|  | Left supplementary motor area (6) | -6 | 10 | 72 | 4.45 | 60 |
|  | Right insula (38) | 44 | 16 | -8 | 4.45 | 75 |
|  | Right middle frontal gyrus (9) | 36 | 20 | 54 | 4.39 | 41 |
| Copying | Left inferior orbitofrontal gyrus (47) | -46 | 22 | -8 | 5.68 | 129 |
|  | Left middle temporal gyrus (21) | -70 | -34 | -14 | 5.54 | 48 |
|  | Right superior frontal gyrus (8)  Right superior frontal gyrus (8) | 6  12 | 42  34 | 54  62 | 5.30  4.30 | 109 |
|  | Right middle frontal gyrus (9) | 24 | 34 | 42 | 5.28 | 65 |
|  | Left supplementary motor area (6) | -2 | 12 | 72 | 4.91 | 62 |
|  | Right inferior frontal operculum (44) | 56 | 16 | 28 | 4.67 | 199 |
|  | Right middle frontal gyrus (8)  Right middle frontal gyrus (8) | 34  36 | 20  12 | 60  62 | 4.34  4.21 | 96 |

B. Brain regions in which regional cerebral glucose metabolism was decreased in PD patients without progressive micrographia compared to healthy controls.

| Task | Regions (BA) | MNI coordinates | | | t-value | Cluster size |
| --- | --- | --- | --- | --- | --- | --- |
|  |  | x | y | z |  |  |
| Free writing | Left inferior orbitofrontal gyrus (47) | -46 | 24 | -12 | 6.96 | 338 |
|  | Right inferior temporal gyrus (20)  Right inferior temporal gyrus (20) | 70  64 | -42  -18 | -14  -24 | 6.55  4.15 | 883 |
|  | Right inferior frontal operculum (44)  Right middle frontal gyrus (9)  Right inferior frontal triangularis (45) | 50  42  56 | 20  22  32 | 34  52  24 | 6.05  5.56  4.97 | 1643 |
|  | Right middle temporal gyrus (21)  Right inferior parietal lobule (39)  Right angular gyrus (39) | 62  56  44 | -62  -58  -68 | 16  46  38 | 5.54  5.33  4.66 | 2015 |
|  | Left thalamus  Left posterior cingulate cortex (23)  Left thalamus | -12  0  -6 | -4  -34  -30 | 12  28  4 | 5.34  5.39  5.31 | 7377 |
|  | Right inferior orbitofrontal gyrus (47)  Right middle frontal gyrus (10)  Right inferior orbitofrontal gyrus (46) | 50  36  50 | 24  62  48 | -10  6  -10 | 5.13  4.38  3.80 | 644 |
|  | Right medial orbitofrontal cortex (11)  Right rectal gyrus (11) | 4  2 | 68  54 | -8  -24 | 5.12  3.70 | 227 |
|  | Left inferior temporal gyrus (20) | -66 | -40 | -16 | 4.74 | 229 |
|  | Right superior frontal gyrus (9) | 24 | 46 | 38 | 4.46 | 159 |
|  | Left middle temporal gyrus (39)  Left middle occipital gyrus (39)  Left middle temporal gyrus (37) | -46  -46  -60 | -62  -78  -58 | 22  24  16 | 4.44  4.09  4.07 | 1021 |
|  | Left middle frontal gyrus (9)  Left precentral gyrus (44)  Left middle frontal gyrus (9) | -42  -48  -28 | 22  14  24 | 46  34  46 | 4.39  3.61  3.56 | 235 |
|  | Left inferior parietal lobule (40) | -50 | -54 | 52 | 4.38 | 154 |
|  | Left middle frontal gyrus (46) | -46 | 50 | -4 | 4.23 | 90 |
|  | Left superior frontal gyrus (8)  Left superior frontal gyrus (9) | -8  -10 | 36  44 | 54  48 | 3.86  3.66 | 114 |
| Copying | Left inferior orbitofrontal gyrus (47) | -46 | 24 | -12 | 6.35 | 288 |
|  | Right inferior temporal gyrus (20)  Right middle temporal gyrus (21)  Right inferior temporal gyrus (20) | 70  62  66 | -40  -62  -22 | -16  16  -22 | 6.25  5.35  5.12 | 3064 |
|  | Right inferior frontal operculum (44)  Right middle frontal gyrus (9)  Right inferior orbitofrontal gyrus (47) | 54  42  48 | 22  22  22 | 28  52  -10 | 6.14  5.19  4.99 | 2574 |
|  | Left posterior cingulate cortex (29)  Left thalamus  Left thalamus | -6  -11  -6 | -48  -32  -20 | 6  4  6 | 5.97  5.94  5.66 | 8347 |
|  | Right cerebellum crus II  Right cerebellum crus I | 46  52 | -56  -52 | -56  -40 | 4.55  4.38 | 322 |
|  | Left inferior temporal gyrus (20)  Left inferior temporal gyrus (20) | -66  -66 | -40  -38 | -14  -22 | 4.52  4.36 | 207 |
|  | Left middle frontal gyrus (46) | -44 | 50 | -6 | 4.48 | 164 |
|  | Right medial orbitofrontal cortex (11)  Right rectal gyrus (11) | 6  2 | 68  52 | -8  -24 | 4.36  4.07 | 225 |
|  | Left middle temporal gyrus (39)  Left middle temporal gyrus (37)  Left middle occipital gyrus (39) | -48  -60  -46 | -62  -58  -76 | 20  14  24 | 4.24  3.79  3.65 | 385 |
|  | Right superior frontal gyrus (9)  Right superior frontal gyrus (10) | 24  16 | 46  62 | 36  28 | 4.17  3.81 | 173 |
|  | Left superior frontal gyrus (10) | -14 | 62 | 22 | 3.68 | 73 |
|  | Left middle occipital gyrus (19) | -38 | -72 | 38 | 3.60 | 81 |

BA = Brodmann area; MNI = Montreal Neurological Institute.


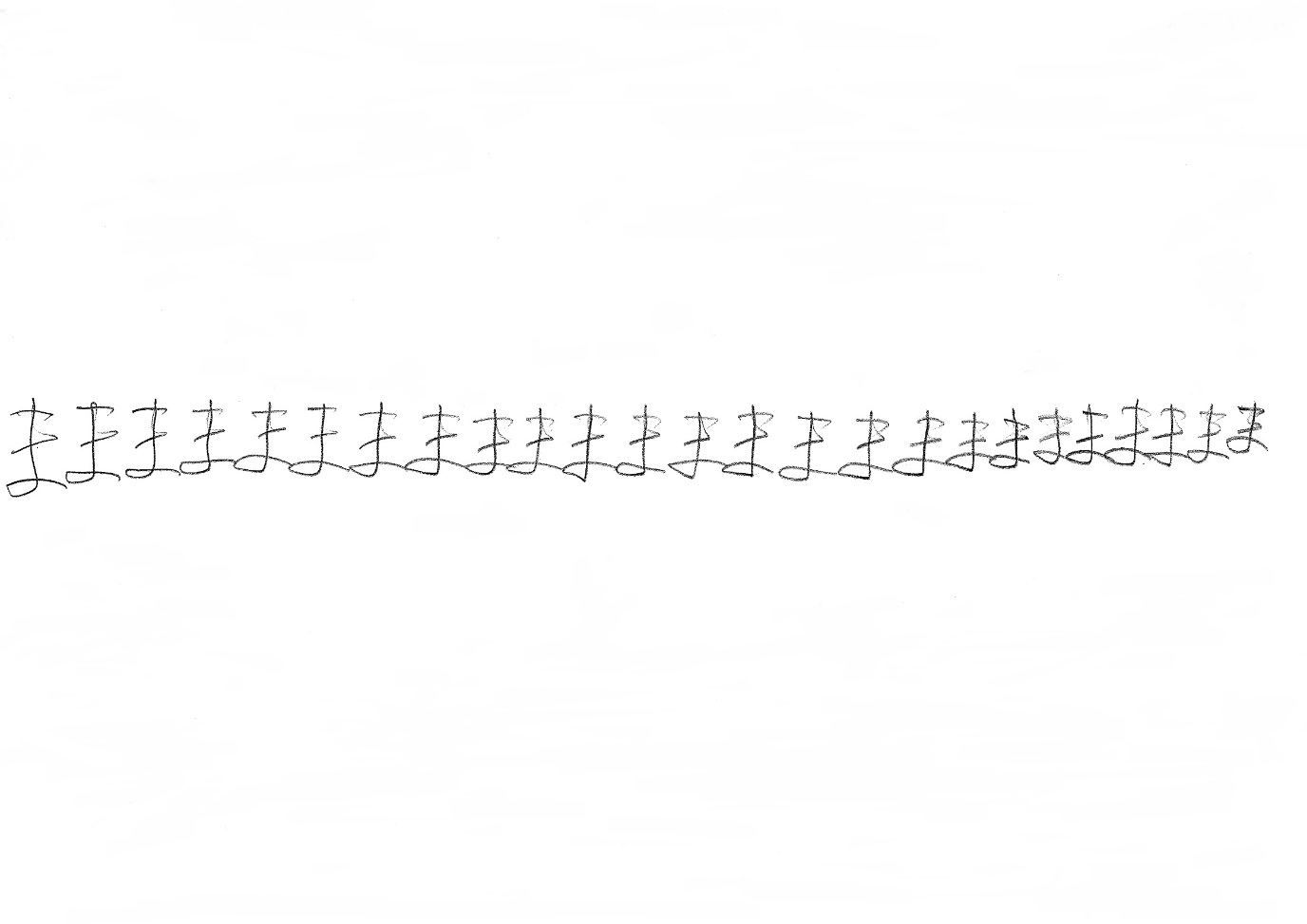


**Supplementary Figure 1.** A sample of progressive micrographia on the free writing task in the healthy controls. In this case, the B value (the slope of the regression line) was -5.56, the Corrected B value (corrected for the mean locus length of the letters) was -3.10, and the p-value was below 0.001. The locus length of the first letter was 69.0 mm, and the mean locus length of the letters was 51.4mm.


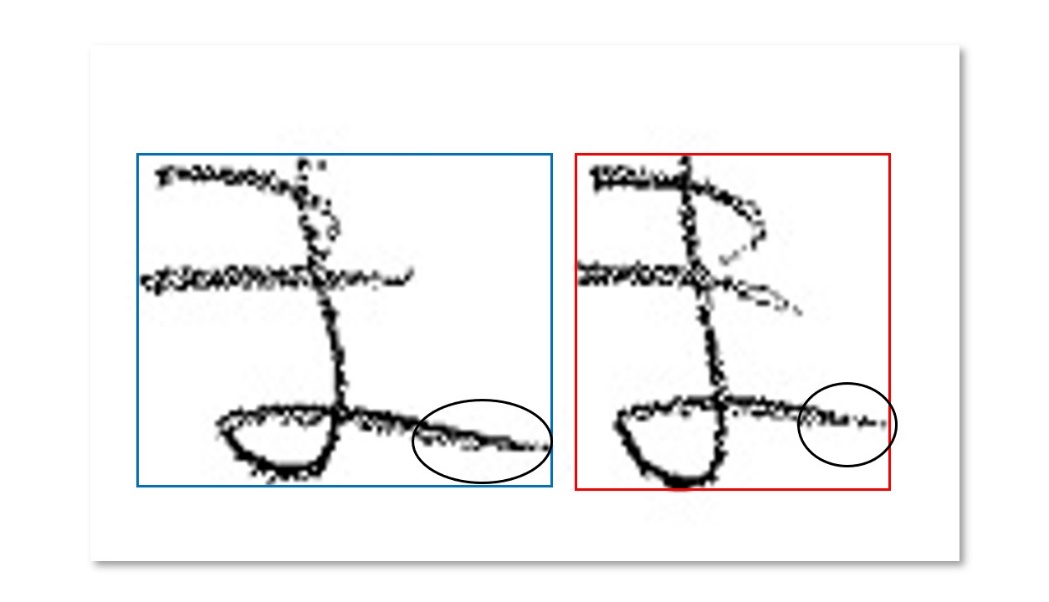


**Supplementary Figure 2.** Samples of “a harai (sweeping)” (black oval), which is one of characteristics of Japanese letter. The area of the rectangle surrounding the letter to the left (blue rectangle) is 1.32 times as large as that to the right (red rectangle). On the other hand, the locus length of the letter to the left is 1.08 times as long as that to the right.
